# Psychological and Organizational Factors Associated With Professional Commitment Among Newly Graduated Psychiatric Nurses: Exploring the Roles of Workplace Anxiety, Spiritual Leadership, and Error Management Climate

**DOI:** 10.1155/jonm/5696980

**Published:** 2026-08-31

**Authors:** Jie Liu, Xuanye Han, Lu Ni, Ping Sun, Zi Yang, Xingyu Zhu, Yuhuan Zhang, Dong Pang

**Affiliations:** ^1^ Second General Surgery Ward, The Second Affiliated Hospital of Harbin Medical University, Harbin, Heilongjiang, China, hrbmush.edu.cn; ^2^ Department of Neurosurgery, The Second Affiliated Hospital of Harbin Medical University, Harbin, Heilongjiang, China, hrbmush.edu.cn; ^3^ Department of Ophthalmology, The Second Affiliated Hospital of Harbin Medical University, Harbin, Heilongjiang, China, hrbmush.edu.cn; ^4^ Department of Rehabilitation, The First Affiliated Hospital of Heilongjiang University of Chinese Medicine, Harbin, Heilongjiang, China, hljucm.edu.cn; ^5^ Harbin Medical University, Harbin, Heilongjiang, China, hrbmu.edu.cn; ^6^ Department of Scientific Research, The Second Affiliated Hospital of Harbin Medical University, Harbin, Heilongjiang, China, hrbmush.edu.cn; ^7^ Department of Student Affairs, The Second Affiliated Hospital of Harbin Medical University, Harbin, Heilongjiang, China, hrbmush.edu.cn; ^8^ Department of Orthopedics, The Second Affiliated Hospital of Harbin Medical University, Harbin, Heilongjiang, China, hrbmush.edu.cn

**Keywords:** error management climate, newly graduated psychiatric nurses, professional commitment, spiritual leadership, workplace anxiety

## Abstract

**Background:**

Newly graduated psychiatric nurses often experience challenges during the transition to professional practice, which may influence their professional commitment. Although workplace anxiety represents an important psychological factor, organizational factors such as spiritual leadership and error management climate may also contribute to variations in professional commitment. However, the relationships among these psychological and organizational factors remain insufficiently understood.

**Objective:**

To examine the associations of workplace anxiety, spiritual leadership, and error management climate with professional commitment among newly graduated psychiatric nurses using generalized additive models (GAMs) and random forest (RF) models to integrate explanatory and predictive analyses.

**Methods:**

A cross‐sectional study was conducted among 264 newly graduated psychiatric nurses recruited from three tertiary psychiatric hospitals in Tianjin, China. Data on workplace anxiety, spiritual leadership, error management climate, and professional commitment were collected using validated questionnaires. A GAM was applied to examine adjusted associations and interaction patterns, while RF analysis was performed to evaluate the predictive contribution of candidate factors.

**Results:**

Error management climate showed a significant positive association with professional commitment in the GAM analysis (*F* = 8.24, *p* = 0.004) and demonstrated the greatest predictive contribution in the RF model according to permutation‐based variable importance. Spiritual leadership did not show a significant main association with professional commitment (*F* = 3.70, *p* = 0.055), but its association with professional commitment varied according to levels of error management climate (Bonferroni‐adjusted *p* = 0.013). Workplace anxiety was not independently associated with professional commitment in the GAM analysis (*F* = 0.39, *p* = 0.533) but showed relatively high predictive contribution in the RF. GAM and RF generated predictions showed strong agreement (*r* = 0.842).

**Conclusion:**

Professional commitment among newly graduated psychiatric nurses is associated with the combined contribution of psychological experiences and organizational conditions. Error management climate demonstrated relatively greater predictive contribution within the RF framework, while workplace anxiety and spiritual leadership may contribute to professional commitment in context‐dependent ways.

**Implications for Nursing Management:**

Nursing managers should consider strengthening error management climate, optimizing leadership practices, and providing psychological support to facilitate professional adaptation and promote professional commitment among newly graduated psychiatric nurses.

## 1. Introduction

Nurses play a crucial role in physical, psychosocial, and spiritual care across various medical settings, including disease prevention, primary care, emergency response, rehabilitation, and health promotion [1]. Nevertheless, the global nursing workforce continues to face challenges in recruiting sufficient nurses. Rapid population aging, increasing healthcare demands, and the uneven distribution of nursing resources have contributed to persistent shortages of qualified nurses worldwide [2]. By 2030, a deficit of around 4.8 million nurses is anticipated, particularly affecting Africa, Southeast Asia, and the WHO Eastern Mediterranean region [2]. This issue is further exacerbated by an aging nursing workforce, high turnover rates, and increasing professional burnout. This situation jeopardizes the quality of healthcare delivery and patient safety [3].

In China, mental and substance use disorders represent a substantial public health challenge. In 2019, these disorders accounted for 20.29 million and 5.76 million disability‐adjusted life years (DALYs), respectively, highlighting their considerable contribution to disease burden [4]. For new nurses in psychiatric departments, the formation of their professional commitment is facing more complicated challenges.

The future requirements for psychiatric nursing and healthcare professions can be described as highly complex. The reasons for this are, for example, the increasing number of multimorbid and chronically mentally ill people, the demand for evidence‐based nursing interventions, and structural changes such as cross‐sectoral collaboration and enhanced interdisciplinary healthcare delivery [5].

Newly graduated nurses may encounter substantial hurdles throughout their transition into professional responsibilities, especially in the rigorous field of mental health nursing. People with mental illnesses sometimes have a lot of different and serious ailments, some of which may be related to other conditions. This condition requires a high dependence on evidence‐based nursing interventions, which makes it much harder for new nurses to adjust [6–8].

Transition shock refers to the challenges and difficulties that newly graduated nurses face during their first year as they transition from students to practicing professionals [9]. A multicenter study involving 3342 new nurses from China showed that transition shock remained a common challenge during early professional development [10]. Facilitating a smooth transition is therefore essential for retaining newly graduated nurses and maintaining a stable nursing workforce [11, 12].

A major mental health problem that new nurses face as they adjust to their new jobs is workplace anxiety. Workplace anxiety, referred to as occupational anxiety or work‐related anxiety, can either impair or enhance job performance [13–15]. Studies demonstrate that increased workplace anxiety is significantly associated with professional burnout, intentions to resign, and job satisfaction [16–18]. In the newly graduated nurse population, workplace anxiety may impair professional flexibility and professional commitment by undermining self‐efficacy and psychological resilience [19]. In the challenging domain of psychiatric nursing, these adverse outcomes may be particularly salient and warrant targeted investigation. However, empirical studies examining such effects among newly graduated psychiatric nurses remain limited.

Leadership is regarded as a crucial external factor in managing workplace stress and fostering employee commitment. Spiritual leadership can establish an organizational vision and values based on individual and team connections and combine their attitudes, values, and behaviors to make employees feel the importance of their work, as well as appreciation and understanding of the organization [20]. It can enhance subordinates’ intrinsic motivation and feeling of career significance, facilitate emotional connections with colleagues, foster a sense of belonging within the organization, and so promote professional socialization [21, 22]. However, most research has focused on Western settings or general nursing settings, resulting in limited understanding of the leadership needs and organizational contexts of newly graduated psychiatric nurses in high‐stress work environments [23, 24].

The organizational atmosphere significantly influences professional commitment, alongside leadership. Error management climate emphasizes an organization’s efficacy in addressing mistakes, including learning from them, preemptively resolving issues, and acknowledging human fallibility [25–28]. Research indicates that a constructive error management climate enhances nursing practices, fosters innovation, and increases employee commitment to their professions [26, 27]. The influence of an error management climate may concurrently be affected by leadership style and individual psychological characteristics. In psychiatric nursing, these complex interaction mechanisms are insufficiently examined through systematic research [26].

Professional commitment refers to an individual’s dedication to and acceptance of the values upheld by their chosen profession [29]. It is closely associated with professional development, nurse retention, and the quality of nursing care [30, 31]. A recent systematic review identified four key contributors to professional commitment: readiness to work, acquiring and maintaining professional knowledge, belief in the profession’s objectives and principles, and ongoing career aspirations [32].

However, the relationships among workplace anxiety, spiritual leadership, error management climate, and professional commitment may not be fully explained by independent additive effects. Workplace anxiety represents an individual psychological challenge, whereas spiritual leadership and error management climate represent organizational and relational resources that may facilitate nurses’ professional adaptation and identification with the nursing profession [33]. From the perspectives of social exchange theory and perceived organizational support, employees’ commitment may be shaped not only by individual experiences but also by the extent to which they perceive organizational recognition and meaningful workplace relationships [34, 35]. Therefore, examining only separate linear associations may overlook the potential interplay among workplace anxiety, spiritual leadership, and error management climate in shaping professional commitment among psychiatric newly graduated nurses.

To address this gap, the present study adopted an integrated explanatory and predictive framework combining generalized additive model (GAM) and random forest (RF) regression. GAM was selected to characterize potentially complex associations and interaction patterns while avoiding restrictive assumptions regarding the functional forms of predictor–outcome relationships [36, 37]. RF regression was subsequently incorporated as a complementary predictive approach to evaluate the relative contribution and robustness of candidate predictors from a machine‐learning perspective.

Therefore, this study aimed to investigate the associations and potential interactions among workplace anxiety, spiritual leadership, error management climate, and professional commitment and to identify variables with greater predictive contributions for professional commitment among Chinese psychiatric newly graduated nurses. The findings may provide evidence for nurse managers to optimize transition programs, strengthen supportive leadership practices, and improve workforce retention.

## 2. Methods

### 2.1. Research Subjects

This research utilized a cross‐sectional methodology. The study was conducted using convenience sampling in three tertiary psychiatric institutions located in Tianjin. The subjects of the research were newly graduated nurses employed in psychiatric departments.

Prior research has commonly characterized newly graduated nurses as individuals in the early stages of clinical practice who are still undergoing role transition from students to professional nurses [9]. Given the complex conditions of patients and the heightened psychological stress within psychiatric care settings, the process of role adaptation and professional socialization may be relatively extended for novice psychiatric nurses. This study defined newly graduated psychiatric nurses as individuals possessing less than three years of clinical experience following graduation, based on an analysis of existing categories.

The eligibility criteria were (1) fewer than 3 years of clinical experience postgraduation, (2) formal registration at the current institution as a clinical nurse, and (3) voluntary participation in the study with signed informed consent.

The exclusion criteria comprised (1) unavailability for further study or full‐time training during the survey duration and (2) being on medical leave, maternity leave, or prolonged leave.

### 2.2. Sample Size Calculation

Sample size considerations were based primarily on recommendations for multivariable regression modeling rather than formulas intended solely for estimating a population mean. According to Green’s rule [38], a minimum sample size of *N* ≥ 50 + 8 m is recommended for evaluating the overall regression model, where *m* represents the number of independent variables. In the present study, eight independent variables were included, yielding a minimum recommended sample size of 114 participants.

A total of 264 newly graduated psychiatric nurses were ultimately included, corresponding to more than twice the minimum recommended sample size.

Because the present study additionally employed GAM and machine‐learning approaches, sample adequacy was further considered in relation to model complexity and potential overfitting. The effective degrees of freedom (EDFs) of the final GAM were relatively low, indicating that the model remained parsimonious despite permitting nonlinear associations and interaction effects.

For the RF analyses, no universally accepted sample size criterion exists. Recent methodological studies have emphasized that sample size requirements depend on model complexity, predictor dimensionality, and the validation strategy employed rather than on a single numerical threshold [39].

To evaluate model robustness, repeated 10‐fold cross‐validation was performed during RF hyperparameter tuning, and bootstrap resampling with 500 iterations was conducted to assess the stability of model performance and the consistency between explanatory and predictive frameworks. These internal validation procedures demonstrated stable model performance and suggested that substantial overfitting was unlikely.

The final sample size was also comparable to or larger than those used in previous cross‐sectional nursing studies examining psychosocial and occupational outcomes among healthcare professionals [40, 41].

### 2.3. Research Instruments

#### 2.3.1. General Information Questionnaire

The researchers developed this questionnaire to obtain fundamental information regarding the demographics and employment history of newly graduated psychiatric nurses. The inquiry mostly pertains to the nurse’s gender, age, only‐child status, marital status, and highest educational attainment. Only‐child status was included as a demographic covariate because family structure may reflect differences in early‐life social environments, psychological characteristics, and coping resources, which may contribute to variations in psychological adaptation and occupational attitudes during the early career stage of nurses [42–44].

#### 2.3.2. Scale for Professional Commitment

The Professional Commitment Scale, originally developed by Blau [45] and subsequently adapted by Pei [46] for the Chinese cultural context, is used to assess the level of professional commitment among newly graduated psychiatric nurses. This scale consists of 24 items divided into five categories: Affective Commitment (6 items), Normative Commitment (5 items), Economic Cost Commitment (4 items), Emotional Cost Commitment (5 items), and Opportunity Commitment (4 items). Notably, four of the items are scored inversely. Each item is rated on a 5‐point Likert scale ranging from 1 (“*strongly disagree*”) to 5 (“*strongly agree*”). The aggregate score ranges from 24 to 120. Elevated scores indicate a greater commitment to the profession. Yang et al. [47] reported a Cronbach’s alpha coefficient of 0.941 for this scale, while the current investigation revealed an overall Cronbach’s *α* coefficient of 0.937, indicating excellent internal consistency. The construct validity of the scale was supported by a Kaiser–Meyer–Olkin (KMO) value of 0.960 and a significant Bartlett’s test of sphericity (*χ* ^2^ = 2572.927, *p* < 0.001).

#### 2.3.3. Scale for Workplace Anxiety

The Workplace Anxiety Scale, developed by McCarthy et al. [48] and subsequently modified by Huang Yafu et al. [49], assesses the anxiety levels of newly graduated psychiatric nurses. This scale comprises eight items and a single dimension. Each item is rated on a 5‐point Likert scale ranging from 1 (“*strongly disagree*”) to 5 (“*strongly agree*”). The total score ranges from 8 to 40. Increased points correlate with heightened anxiety in the workplace. McCarthy et al. [48] reported a Cronbach’s alpha coefficient of 0.94, whereas the current study yielded a Cronbach’s *α* coefficient of 0.887, indicating good internal consistency. The scale also demonstrated satisfactory construct validity, with a KMO value of 0.919 and a significant Bartlett’s test of sphericity (*χ* ^2^ = 890.190, *p* < 0.001).

#### 2.3.4. Scale for Spiritual Leadership

The revised Spiritual Leadership Scale developed by Yang Zhenfang et al. [50] is used to evaluate how newly graduated psychiatric nurses perceive the quality of spiritual leadership within their departments. This scale consists of 14 items, which are divided into three categories: Vision Building (5 items), Faith (5 items), and Altruistic Love (4 items). Each item is rated on a 6‐point Likert scale ranging from 1 (“*strongly disagree*”) to 6 (“*strongly agree*”). The overall score can range from 14 to 84. A higher score indicates a greater perception of spiritual leadership. Prior research indicated a Cronbach’s *α* coefficient of 0.813 among young nurses [51]. This study reports an overall Cronbach’s *α* coefficient of 0.889, indicating excellent internal consistency. The construct validity was supported by a KMO value of 0.925 and a significant Bartlett’s test of sphericity (*χ* ^2^ = 1222.766, df = 91, *p* < 0.001).

#### 2.3.5. Scale for Assessing Error Management Climate

The updated Error Management Climate Scale by Van Dyck et al. [26] assesses the perceived error management environment at the unit level among newly graduated psychiatric nurses. This measure comprises four components: Error Learning (4 items), Error Thinking (3 items), Error Competence (5 items), and Error Communication (4 items). The scale consists of 16 items in total. Each item is rated on a 5‐point Likert scale ranging from 1 (“*strongly disagree*”) to 5 (“*strongly agree*”). The total score range is from 16 to 80. A higher score indicates a more favorable perception of the error management climate. Previous studies suggest that this scale exhibits substantial reliability among nurses, as indicated by a Cronbach’s *α* coefficient of 0.924 [52]. In the present study, the scale demonstrated good internal consistency, with an overall Cronbach’s *α* coefficient of 0.864. The construct validity was supported by a KMO value of 0.894 and a significant Bartlett’s test of sphericity (*χ* ^2^ = 1775.370, *p* < 0.001).

### 2.4. Methods for Conducting Surveys

This study employed an online survey through Wenjuanxing, with standardized instructions and completion protocols inside the questionnaire. Before initiating the main survey, 15 newly graduated psychiatric nurses who met the inclusion criteria were randomly selected from the researcher’s hospital for a trial survey. The questionnaire required 4–7 min to complete, which was deemed acceptable. Subsequent to the pilot survey, the researcher transmitted the QR code and the study’s objective to the chief nurses or nursing department directors at two other hospitals. The management departments approved the survey before it was executed. Upon completion, the questions were submitted, and each IP address was permitted to participate only once.

Before data collection, predefined criteria were established to identify invalid responses. Questionnaires completed in less than 2 min were considered invalid because this duration was regarded as insufficient for carefully reading and answering all items. In addition, questionnaires with identical responses across all items were excluded as low‐quality responses.

A total of 290 questionnaires were received. After excluding 26 invalid questionnaires based on the predefined criteria, 264 valid responses were retained, resulting in an effective response rate of 91.03%.

This study was reported in accordance with the Strengthening the Reporting of Observational Studies in Epidemiology (STROBE) guidelines for cross‐sectional studies.

### 2.5. Statistical Methods

#### 2.5.1. Study Variables and Descriptive Analysis

The primary variables included workplace anxiety, spiritual leadership, error management climate, and professional commitment. Demographic variables comprised age, gender, only‐child status, marital status, and educational attainment. All categorical variables were converted into factors prior to analysis. Continuous variables were summarized using means and standard deviations (SDs), whereas categorical variables were reported as frequencies and percentages.

Data completeness was verified prior to analysis, and no missing data imputation was required.

#### 2.5.2. GAM

GAM was applied as the primary inferential approach to examine adjusted associations among workplace anxiety, spiritual leadership, error management climate, and professional commitment. Professional commitment was treated as a continuous outcome variable and modeled using a Gaussian distribution with an identity link function.

Thin‐plate regression splines (bs = “tp”) were used to estimate potential nonlinear patterns of the three core predictors. Tensor‐product smooths “te()” were applied to examine prespecified pairwise interaction effects among workplace anxiety, spiritual leadership, and error management climate. Age, sex, only‐child status, marital status, and educational attainment were included as linear covariates.

The GAM was fitted using restricted maximum likelihood (REML). The basis dimension was initially set at *k* = 4 for all smooth terms. Smoothing parameters were estimated automatically through REML optimization. The adequacy of basis dimensions was assessed using gam.check(), including the k‐index diagnostic.

Model adequacy was further evaluated using graphical residual diagnostics (residual‐versus‐fitted plots, Q–Q plots, residual histograms, and observed‐versus‐fitted plots) and concurvity analyses to assess potential model misspecification and redundancy among smooth terms.

Model performance was assessed using adjusted *R* ^2^, explained deviance, and REML score. EDFs, F statistics, and *p* values were used to evaluate smooth terms. Because three tensor‐product interaction terms were examined simultaneously, Bonferroni correction was applied to interaction *p* values. This correction was restricted to interaction terms because these analyses represented the prespecified multiple interaction hypotheses, whereas marginal smooth terms were interpreted as adjusted associations within the GAM framework. For descriptive purposes, locations where the estimated smooth functions crossed the model reference level were reported to facilitate graphical interpretation of the fitted partial effects.

Wald‐type 95% confidence intervals for parametric coefficients were calculated based on the estimated covariance matrix of the fitted GAM.

#### 2.5.3. RF Analysis

RF regression was conducted as a complementary predictive approach to evaluate the relative contribution of predictors under a nonparametric machine‐learning framework. Unlike GAM, which estimates adjusted associations, RF focuses on predictive performance and variable importance without prespecifying functional relationships.

The RF was implemented using the ranger algorithm within the caret framework in R. Hyperparameters, including the number of variables randomly sampled at each split (mtry) and terminal node size (min.node.size), were optimized using repeated 10‐fold cross‐validation with five repetitions. The final model, trained using the optimal hyperparameters and 500 trees, was subsequently evaluated using cross‐validated *R* ^2^, root mean squared error (RMSE), and mean absolute error (MAE).

Permutation‐based variable importance, interpreted as predictive contribution within the RF model, was quantified as the mean increase in prediction error (mean squared error, MSE) after randomly permuting the values of each predictor while keeping all other predictors unchanged. The procedure was repeated 30 times for each predictor, with larger increases in MSE indicating greater predictive contribution. This metric reflects the relative usefulness of each variable for prediction within the RF model rather than an independent effect, causal influence, or underlying mechanism of professional commitment.

Partial dependence plots (PDPs) were generated to visualize the marginal predictive patterns between the core predictors and professional commitment derived from the fitted RF. These plots were interpreted descriptively as average marginal prediction patterns and not as evidence of causal effects.

#### 2.5.4. Model Consistency and Bootstrap Stability Analysis

To examine consistency between explanatory and predictive frameworks, predicted professional commitment values generated from the GAM and RF were compared using Pearson correlation coefficients.

Bootstrap resampling was performed to evaluate internal model stability. Five hundred bootstrap iterations were conducted to assess both the stability of GAM explanatory performance and the consistency between GAM and RF predictions. Bootstrap confidence intervals were calculated using the percentile method. These analyses were interpreted as indicators of model robustness rather than evidence of causal inference.

#### 2.5.5. Statistical Software

All statistical analyses were conducted using R software Version 4.3.0. The principal packages included readxl, dplyr, psych, mgcv, gratia, caret, ranger, iml, ggeffects, ggplot2, patchwork, openxlsx, and boot. All statistical tests were two‐sided, and *p* < 0.05 was considered statistically significant.

## 3. Results

### 3.1. Demographic Characteristics and Descriptive Statistics

A total of 264 newly graduated psychiatric nurses were included in the final analysis. The mean age of participants was 25.83 ± 2.09 years. Among the participants, 167 (63.26%) were female and 97 (36.74%) were male. More than half of the participants were only children (54.55%), and 147 (55.68%) were married. Most participants had a bachelor’s degree or below (84.09%).

The descriptive statistics of the main study variables are presented in Table 1. The mean scores were 51.14 ± 10.65 for spiritual leadership, 28.86 ± 7.02 for workplace anxiety, 92.27 ± 16.39 for professional commitment, and 60.53 ± 10.62 for error management climate.

**TABLE 1:** Demographic characteristics and key study variables of newly graduated psychiatric nurses (*n* = 264).

Variable	Category/statistic	*n* (%)/mean (SD)
Demographic characteristics		
Gender	Male	97 (36.74%)
	Female	167 (63.26%)
Age (years)		25.83 (2.09)
Only child	Yes	144 (54.55%)
	No	120 (45.45%)
Marital status	Unmarried	117 (44.32%)
	Married	147 (55.68%)
Highest educational attainment	Bachelor or below	222 (84.09%)
	Master or above	42 (15.91%)
Key Study Variables		
Spiritual leadership		51.14 (10.65)
Workplace anxiety		28.86 (7.02)
Professional commitment		92.27 (16.39)
Error management climate		60.53 (10.62)

### 3.2. GAM Analysis

#### 3.2.1. Model Performance and Diagnostic Evaluation

A GAM was constructed to examine the associations of workplace anxiety, spiritual leadership, and error management climate with professional commitment while adjusting for demographic covariates, including age, gender, only‐child status, marital status, and educational level.

The GAM demonstrated moderate explanatory performance, explaining 45.5% of the deviance in professional commitment (adjusted *R* ^2^ = 0.419, REML = 1021.88).

**FIGURE 1:**
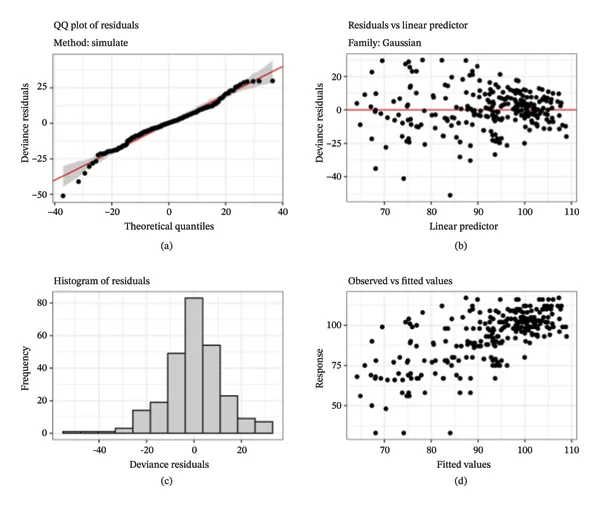
Diagnostic plots of the GAM predicting professional commitment. Note: Diagnostic plots assessing the adequacy of the GAM predicting professional commitment. (a) Residuals versus fitted values assessing potential systematic patterns; (b) normal Q–Q plot evaluating the approximate distributional characteristics of residuals; (c) histogram of deviance residuals showing residual distribution characteristics; and (d) observed versus fitted values illustrating the correspondence between observed and model‐estimated professional commitment scores. The diagnostic evaluation did not indicate substantial model misspecification or residual problems.

The k‐index diagnostics did not suggest inadequate basis dimensions (k‐index range: 0.92–1.02; all *p* values > 0.05), indicating that the selected basis dimensions were sufficient for representing the fitted smooth functions. Residual diagnostics showed an approximately symmetric distribution without evident systematic deviations across fitted values. Concurvity diagnostics indicated no substantial redundancy among smooth terms, suggesting that the estimated associations were not markedly affected by excessive dependency among predictors (Figure 1).

#### 3.2.2. Main Effects of Psychological and Organizational Factors on Professional Commitment

The estimated smooth terms from the GAM are summarized in Table 2. Among the three focal predictors, error management climate showed a significant positive marginal association with professional commitment (*F* = 8.24, *p* = 0.004). In contrast, the smooth terms for spiritual leadership and workplace anxiety did not reach statistical significance (spiritual leadership: *F* = 3.70, *p* = 0.055; workplace anxiety: *F* = 0.39, *p* = 0.533).

**TABLE 2:** Smooth terms and interaction effects estimated by the GAM.

Term	EDF	*F*	*p* *-*value	Bonferroni‐adjusted *p*‐value
s (workplace anxiety)	1	0.39	0.533	0.533
s (spiritual leadership)	1	3.70	0.055	0.055
s (error management climate)	1	8.24	0.004	0.004
te (workplace anxiety × spiritual leadership)	3.55	2.71	0.033	0.099
te (workplace anxiety × error management climate)	1	0.37	0.542	1
te (spiritual leadership × error management climate)	3.94	3.81	0.004	0.013

*Note:* Bonferroni correction was applied to the three tensor‐product interaction terms. EDF values close to 1 indicate approximately linear associations. Tensor‐product smooths were used to evaluate prespecified pairwise interactions.

The values of the EDFs for all three univariate smooth terms were approximately 1.00, indicating that the associations of workplace anxiety, spiritual leadership, and error management climate with professional commitment were largely linear within the observed data range. The parametric coefficients of demographic covariates are presented in Supporting Table S1. No statistically significant associations were observed between demographic characteristics and professional commitment (all 95% CIs included zero).

**FIGURE 2:**
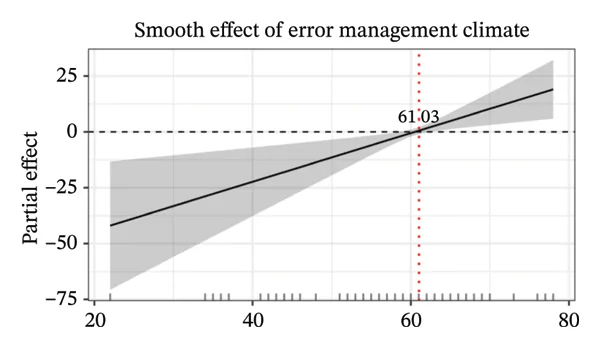
Estimated partial effect of error management climate on professional commitment derived from the GAM. Note: The figure presents the estimated partial effect of error management climate on professional commitment obtained from the GAM. The solid black line represents the estimated smooth function, and the shaded area represents the 95% confidence interval. The horizontal dashed line indicates a partial effect of zero. The vertical dotted line indicates the zero‐crossing location (61.03 points), representing the point where the estimated partial effect intersects the model reference level. This value should not be interpreted as a managerial threshold or intervention cutoff.

Figure 2 presents the estimated partial effect of error management climate on professional commitment. The smooth function demonstrated an approximately linear positive pattern, indicating that higher levels of error management climate were generally associated with higher professional commitment scores. The fitted curve crossed the GAM reference level at approximately 61.03 points, suggesting that scores above this value were associated with above‐average predicted professional commitment. This point reflects the location of the fitted smooth function relative to the model reference level and should not be interpreted as a clinical threshold or recommended cutoff value.

#### 3.2.3. Interaction Effects Between Psychological and Organizational Factors

Three prespecified tensor‐product interaction terms were examined (Table 2). After Bonferroni correction for multiple interaction testing, only the interaction between spiritual leadership and error management climate was statistically significant (EDF = 3.94, *F* = 3.81, adjusted *p* = 0.013).

The interaction between workplace anxiety and spiritual leadership was significant before correction (unadjusted *p* = 0.033) but was not statistically significant after Bonferroni adjustment (adjusted *p* = 0.099). No evidence of interaction was observed between workplace anxiety and error management climate (unadjusted *p* = 0.542; adjusted *p* = 1.000).

**FIGURE 3:**
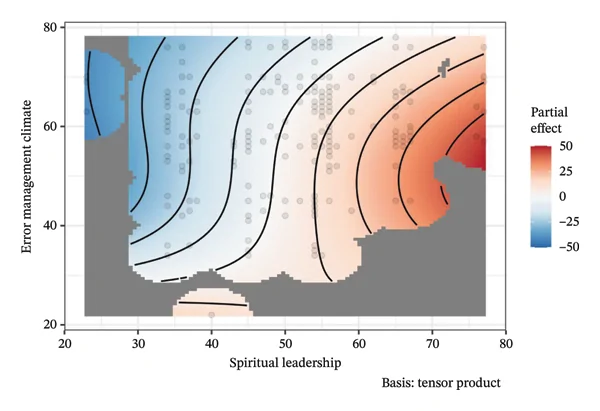
Tensor‐product interaction between spiritual leadership and error management climate in relation to professional commitment. Note: Two‐dimensional tensor‐product smooth visualization of the interaction between spiritual leadership and error management climate estimated by the GAM. The color gradient represents model‐estimated professional commitment scores, with cooler colors indicating relatively lower predicted values and warmer colors indicating relatively higher predicted values. Black contour lines indicate combinations of spiritual leadership and error management climate associated with similar predicted professional commitment levels. Closer contour lines indicate more rapid changes in predicted professional commitment across the predictor range.

As illustrated in Figure 3, the estimated joint effect of spiritual leadership and error management climate on professional commitment varied across their observed combinations. Specifically, the tensor‐product smooth indicated that the predicted professional commitment was not determined by either factor alone but varied according to their combined levels. Higher predicted professional commitment scores were primarily observed in regions where higher values of spiritual leadership and error management climate co‐occurred within the observed data range.

An exploratory analysis was additionally conducted to examine whether spiritual leadership modified the association between workplace anxiety and professional commitment. Although the estimated interaction showed a potential pattern of variation across levels of spiritual leadership (Supporting Figure S1), this interaction did not remain statistically significant after Bonferroni correction (adjusted *p* = 0.099). Therefore, this finding was considered exploratory and was not interpreted as confirmatory evidence of moderation.

### 3.3. RF Analysis

#### 3.3.1. Predictive Performance

The optimal tuning parameters identified through repeated 10‐fold cross‐validation were mtry = 4 and minimum node size = 5. The RF demonstrated good predictive performance, with a cross‐validated *R* ^2^ of 0.462, RMSE of 12.31, and MAE of 9.09.

#### 3.3.2. Permutation‐Based Predictive Contribution

**FIGURE 4:**
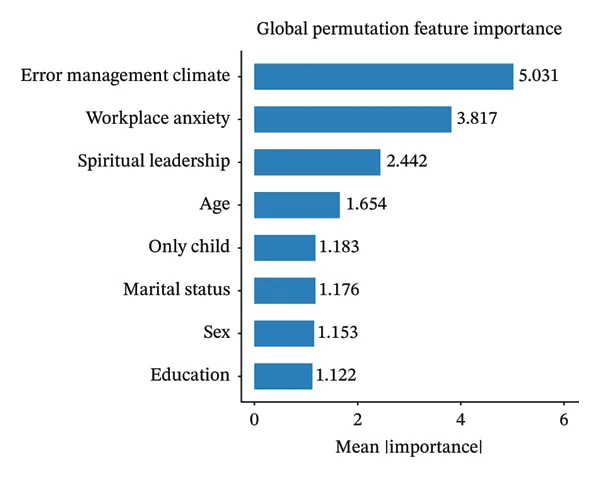
Predictive contribution ranking derived from the RF. Note: Predictive contribution ranking derived from the RF. The *x*‐axis represents the mean increase in prediction error after randomly permuting each predictor. Larger values indicate a greater contribution to predictive performance, suggesting greater relative predictive contribution within the RF model. Error management climate, workplace anxiety, and spiritual leadership demonstrated relatively higher RF‐based predictive contributions among the examined variables. These rankings represent model‐specific predictive utility and should not be interpreted as evidence of causal importance or independent determinants of professional commitment.

Permutation‐based predictive contribution analysis showed that error management climate showed the highest relative predictive contribution within the RF model, followed by workplace anxiety and spiritual leadership, whereas demographic variables demonstrated comparatively smaller contributions (Supporting Table S2). Figure 4 presents the ranking of permutation‐based predictive contributions. Although workplace anxiety was not statistically significant in the GAM marginal analysis, it demonstrated relatively high predictive contribution in the RF.

### 3.4. Partial Dependence Analysis

**FIGURE 5:**
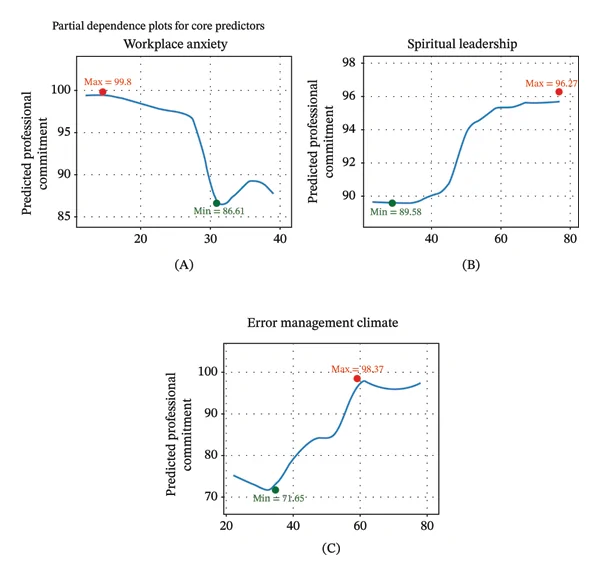
Partial dependence plots of core predictors for professional commitment. Note: Partial dependence plots illustrating the model‐estimated predictive relationships between the core predictors and professional commitment from the RF. (A) Workplace anxiety. (B) Spiritual leadership. (C) Error management climate. The solid line represents the estimated partial dependence function, and annotated points indicate approximate locations of the highest and lowest predicted values within the observed predictor range. The *y*‐axis represents model‐predicted professional commitment values. These plots illustrate average model‐based prediction patterns while holding other variables constant and should not be interpreted as evidence of independent effects, causal relationships, or clinically meaningful thresholds.

Figure 5 illustrates the partial dependence patterns of workplace anxiety, spiritual leadership, and error management climate with respect to predicted professional commitment.

For workplace anxiety (Figure 5A), predicted professional commitment values remained relatively stable at lower anxiety levels but showed a decreasing pattern at higher levels within the observed data range, indicating a negative predictive pattern.

For spiritual leadership (Figure 5B), predicted professional commitment values remained relatively stable at lower levels, increased across the intermediate range, and subsequently plateaued at higher levels, indicating a positive but nonlinear predictive pattern.

For error management climate (Figure 5C), predicted professional commitment values increased across the middle‐to‐high range and stabilized at higher levels, indicating a positive predictive pattern.

The magnitude of predictive variation, quantified as the range of predicted professional commitment values across the observed predictor range (maximum minus minimum partial dependence), was 26.72 for error management climate, 13.18 for workplace anxiety, and 6.69 for spiritual leadership. These patterns were broadly consistent with the relative ranking observed in the permutation importance analysis.

### 3.5. Model Agreement and Stability Analysis

**FIGURE 6:**
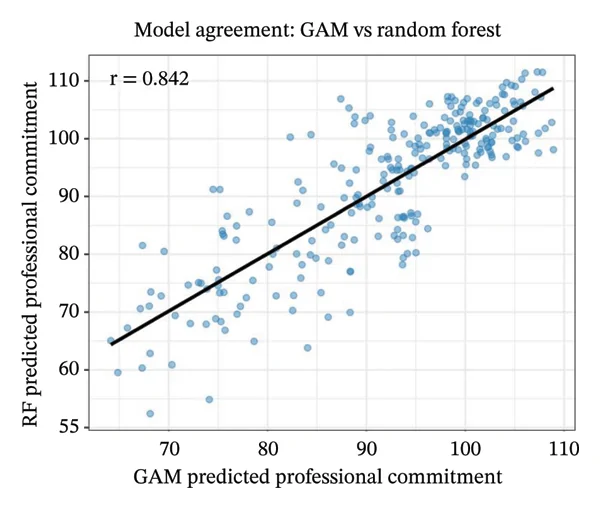
Agreement between GAM and RF predictions of professional commitment. Note: Scatterplot showing the relationship between predicted professional commitment values generated by the GAM and RF models. Each point represents an individual participant. The solid line represents the linear regression line between the two sets of predicted values. The Pearson correlation coefficient reflects the degree of association between predictions generated by the two analytical approaches.

Predicted professional commitment values generated from the GAM and RF showed a strong correlation (Pearson *r* = 0.842), indicating good consistency between the two analytical approaches (Figure 6).

Bootstrap analyses demonstrated stable model performance. The adjusted *R* ^2^ of the GAM remained within a 95% bootstrap confidence interval of 0.375–0.599. Moreover, the correlation between GAM and RF generated predictions remained consistent across bootstrap samples, with a 95% confidence interval of 0.745–0.875.

## 4. Discussion

This study examined the associations of workplace anxiety, spiritual leadership, and error management climate with professional commitment among newly graduated psychiatric nurses using complementary explanatory and predictive approaches. Four principal findings emerged.

First, error management climate demonstrated a significant positive association with professional commitment in the GAM analysis and showed the highest predictive contribution within the RF. Second, spiritual leadership was not significantly associated with professional commitment in the GAM analysis. However, the significant interaction between spiritual leadership and error management climate suggested that the relationship between spiritual leadership and professional commitment may differ depending on levels of error management climate. Third, although workplace anxiety was not significantly associated with professional commitment after adjustment in the GAM, it demonstrated notable predictive contribution in the RF. Finally, the strong agreement between GAM and RF generated predictions suggested that the explanatory and predictive approaches identified broadly consistent patterns, providing complementary perspectives on factors associated with professional commitment. Together, these findings suggest that individual psychological experiences and organizational conditions were associated with variations in professional commitment among newly graduated psychiatric nurses.

### 4.1. Error Management Climate and Professional Commitment

The present study found that error management climate showed a significant positive association with professional commitment in the GAM analysis (*F* = 8.24, *p* = 0.004) and demonstrated the highest predictive contribution within the RF model.

This finding highlights the potential role of error management climate in supporting newly graduated psychiatric nurses’ professional attitudes and adaptation to clinical practice. Previous studies have demonstrated that a high error management climate is characterized by shared norms that encourage employees to communicate about errors, analyze their causes, and implement corrective actions, thereby helping organizations reduce the negative consequences of mistakes [26]. Moreover, such a climate promotes constructive responses to errors and experiential learning [53]. These characteristics may be particularly relevant for newly graduated psychiatric nurses, who, similar to newly graduated nurses in other clinical settings, may experience uncertainty, concerns regarding clinical competence, and challenges during early professional transition [9].

From the perspective of psychological safety theory, employees are more likely to engage in learning behaviors and interpersonal communication when they perceive that mistakes will not lead to blame or humiliation [54]. Therefore, an error management climate that emphasizes learning‐oriented responses and constructive error discussion may encourage help‐seeking and speaking‐up behaviors, facilitating adaptation and confidence development among novice psychiatric nurses.

Importantly, error management climate demonstrated the RF‐based predictive contribution among all study variables and showed the largest change in model‐predicted professional commitment across its observed range in the partial dependence analysis. Across the observed range of error management climate, the predicted professional commitment score generated by the model varied by approximately 26.7 points, suggesting that differences in organizational error management practices may be associated with variations in model‐estimated professional commitment levels. Given that organizational climate is potentially modifiable, interventions aimed at fostering a nonpunitive and learning‐oriented error management climate may represent a promising approach to supporting professional commitment among newly graduated psychiatric nurses.

However, these findings should be interpreted with appropriate caution. Because RF predictive contribution reflects predictive utility rather than independent statistical effects, the observed predictive contribution of error management climate should not be interpreted as evidence that it is the most influential causal determinant of professional commitment. Instead, the RF findings indicate that error management climate contributed most to prediction within the present dataset, complementing the adjusted associations identified by the GAM.

### 4.2. The Joint Influence of Spiritual Leadership and Error Management Climate

A notable finding of this study was the significant interaction between spiritual leadership and error management climate in relation to professional commitment (EDF = 3.94, *F* = 3.81, Bonferroni‐adjusted *p* = 0.013). Higher predicted professional commitment scores were primarily observed in regions where higher levels of spiritual leadership and error management climate co‐occurred within the observed data range.

This finding suggests that leadership behaviors may not operate independently from the organizational context in which they occur. According to situational strength theory, contextual cues and organizational conditions can shape employees’ perceptions and behavioral responses by influencing the extent to which individual characteristics are expressed in the workplace [55]. When organizational environments encourage learning from mistakes and foster psychological safety, such conditions may provide a supportive context in which nurses can perceive spiritual leaders’ messages regarding meaning, purpose, and professional values as aligned with their workplace experiences [20, 54].

The observed interaction may also be interpreted from the perspective of social exchange theory, which proposes that employees develop reciprocal relationships with organizations based on perceived support and resource exchange [56]. When newly graduated nurses experience supportive leadership and a constructive organizational climate, they may be more likely to perceive that their organization values their contributions and cares about their well‐being, consistent with the concept of perceived organizational support [57]. Similarly, previous nursing research has suggested that supportive leadership and empowering work environments are associated with stronger organizational commitment among nurses [58].

Conversely, in environments characterized by punitive responses to errors or limited organizational support, leadership behaviors may have a more limited association with professional commitment because organizational practices may create a mismatch between leaders’ messages and employees’ workplace experiences. This interpretation is consistent with previous studies, indicating that positive leadership styles are associated with favorable nurse outcomes within supportive organizational contexts [59, 60].

These contextual mechanisms may be particularly relevant for newly graduated psychiatric nurses, who often undergo challenging professional transitions and may experience uncertainty regarding clinical competence during early practice [9]. A supportive error management climate may provide a learning‐oriented environment that facilitates error reflection, help‐seeking, and adaptation by reducing concerns about negative consequences associated with discussing mistakes [26, 54]. In such an environment, leaders’ messages regarding meaning and professional values may be more likely to be perceived as aligned with nurses’ daily workplace experiences [20]. Therefore, the present findings suggest that leadership development initiatives may benefit from being implemented alongside efforts to strengthen organizational cultures that support learning and psychological safety. Rather than focusing solely on leadership training, organizations may consider simultaneously improving workplace climates that enable leaders’ values to be expressed consistently in daily practice.

Finally, the nonsignificant main effect of spiritual leadership should be interpreted in light of the interaction observed in this study. Although the smooth term for spiritual leadership did not reach statistical significance in the adjusted GAM analysis (*F* = 3.70, *p* = 0.055), the RF partial dependence analysis demonstrated that predicted professional commitment varied by approximately 6.69 points across the observed range of spiritual leadership. This pattern suggests that variations in spiritual leadership may still be associated with differences in predicted professional commitment, particularly when accompanied by favorable organizational conditions. Importantly, these patterns should be interpreted as descriptive trends observed within the study sample and should not be considered as definitive thresholds for clinical practice or managerial interventions.

### 4.3. Understanding the Role of Workplace Anxiety

Workplace anxiety did not demonstrate an independent association with professional commitment in the adjusted GAM (*F* = 0.39, *p* = 0.533). However, it emerged as one of the variables with greater permutation‐based predictive contributions in the RF. These findings should not be interpreted to indicate that workplace anxiety is unrelated to professional commitment. Instead, they suggest that the relationship between workplace anxiety and professional commitment may involve complex patterns that are not fully captured by independent adjusted associations alone.

The GAM estimated the independent association between workplace anxiety and professional commitment after accounting for other variables, whereas the RF evaluated the overall contribution of workplace anxiety to prediction. Therefore, workplace anxiety may still provide important information for characterizing factors associated with relatively lower predicted levels of professional commitment, even if its independent association was not statistically significant in the GAM analysis. The relatively high RF importance of workplace anxiety indicates that this variable contributed to the model’s predictive performance when considered together with other variables but does not imply an independent causal influence on professional commitment. The explanatory and predictive findings should therefore be regarded as complementary rather than conflicting because they address different scientific questions.

Previous studies have consistently demonstrated that anxiety and work‐related stress are associated with burnout, lower job satisfaction, and stronger intentions to leave the profession [61, 62]. However, according to the Job Demands–Resources model, the adverse effects of psychological demands may be mitigated when employees have access to sufficient organizational resources [63].

The present findings are consistent with this perspective and suggest that supportive organizational conditions, including positive leadership and a constructive error management climate, may provide supportive contexts associated with more favorable professional outcomes among nurses experiencing workplace anxiety. Consequently, the absence of an independent association in the GAM should not be interpreted as evidence that workplace anxiety is unimportant. Rather, workplace anxiety remains a psychological characteristic with potential predictive utility within the RF framework that may warrant attention when developing supportive interventions for newly graduated nurses during the transition to professional practice.

Exploratory analyses further suggested that the relationship between workplace anxiety and professional commitment may vary across different levels of spiritual leadership. Specifically, the negative pattern appeared weaker at higher levels of spiritual leadership (Supporting Figure S1). However, the workplace anxiety × spiritual leadership interaction did not remain statistically significant after Bonferroni correction (adjusted *p* = 0.099). Therefore, this pattern should be interpreted cautiously as an exploratory observation rather than confirmatory evidence of moderation, and further studies are needed to determine whether organizational resources can buffer the potential adverse influence of workplace anxiety.

Finally, the strong agreement between the predictions generated by the GAM and RF models (*r* = 0.842) indicates that the two analytical approaches produced broadly consistent conclusions despite addressing different objectives. The convergence of findings across explanatory and predictive analyses therefore strengthens confidence in the consistency of the findings across analytical approaches and provides complementary insights for both theory development and nursing management practice.

## 5. Implications for Nursing Management

This study has several implications for nursing management as follows.

First, creating a positive error management climate should be considered a priority strategy for improving professional commitment among newly graduated psychiatric nurses. Encouraging open communication about errors, promoting learning from mistakes, and reducing punitive responses may facilitate professional adaptation and strengthen commitment to the nursing profession.

Second, leadership development initiatives should be implemented together with efforts to improve organizational culture. The findings suggest that spiritual leadership may be most effective when nurses work in environments characterized by psychological safety and supportive responses to errors.

Third, although workplace anxiety did not demonstrate an independent association with professional commitment, its predictive contribution suggests that nursing managers should pay close attention to the psychological well‐being of newly graduated nurses. Transition support programs, mentoring systems, and accessible psychological services may help identify and support nurses who are vulnerable during the early stages of professional practice.

Finally, professional commitment appears to be influenced by multiple interrelated factors. Therefore, interventions simultaneously targeting organizational culture, leadership practices, and psychological support are likely to be more effective than strategies focusing on a single domain.

## 6. Limitations

This study has several shortcomings. First, participants were recruited from three psychiatric institutions inside a single city, which may limit the generalizability of the findings to other geographic regions and healthcare contexts. Future multicenter and multiregional studies are needed to further evaluate the applicability of these findings.

Second, the study relied on self‐reported questionnaires to assess workplace anxiety, spiritual leadership, error management climate, and professional commitment. Although validated instruments were used, self‐reported measures may be affected by recall bias, social desirability bias, and potential common method bias. Future research incorporating multisource assessments or objective organizational indicators may help improve measurement validity.

Third, the cross‐sectional design prevents conclusions regarding temporal relationships and causality. Although the present study identified associations and interaction patterns among psychological and organizational factors, longitudinal or intervention studies are required to further clarify the underlying mechanisms.

Finally, although the sample size was adequate for the planned analyses and internal validation procedures were performed, the complexity of the analytical framework should be acknowledged. The combination of GAM, RF modeling, interaction analyses, and sensitivity analyses was conducted in a relatively modest sample, and external validation in independent cohorts was not available. Future studies using larger samples and externally validated predictive models are needed to confirm the robustness and generalizability of these findings.

## 7. Conclusions

This study provides insights into the factors associated with professional commitment among newly graduated psychiatric nurses using complementary explanatory and predictive approaches. Error management climate showed a significant positive association with professional commitment and demonstrated the greatest predictive contribution to accuracy in the RF model within the present dataset. This finding indicates greater predictive utility of this variable within the RF framework rather than evidence of causal determination. Although spiritual leadership did not show a significant main association in the adjusted GAM analysis, its relationship with professional commitment varied across different levels of error management climate. Workplace anxiety was not independently associated with professional commitment after adjustment but demonstrated predictive relevance within the RF model.

These findings highlight that professional commitment among newly graduated psychiatric nurses is associated with the interplay between individual psychological experiences and organizational conditions during the transition to professional practice. Enhancing error management climate, optimizing leadership practices, and providing psychological support may represent potential management strategies to support professional adaptation and commitment among newly graduated psychiatric nurses.

## Author Contributions

Jie Liu: conceptualization, methodology, investigation, data curation, writing–original draft.

Xuanye Han: methodology, validation, supervision, writing–review and editing.

Lu Ni: investigation, data curation, formal analysis.

Ping Sun: investigation, resources, project administration.

Zi Yang: data curation, software, visualization.

Xingyu Zhu: validation, visualization, writing–review and editing.

Yuhuan Zhang: conceptualization, funding acquisition, writing–review and editing, project administration.

Dong Pang: conceptualization, supervision, writing–review and editing.

## Funding

The Heilongjiang Higher Education Association Special Project on the Third Plenary Session of the 20th CPC Central Committee and the 2024 National Education Conference (24GJZXE003) and the 2025 Harbin Medical University Key Project for Comprehensive Reform and Quality Enhancement in Ideological and Political Work (HYDSZZDXM011) provided funding for this study.

## Disclosure

The funders had no role in the study design; data collection, analysis, and interpretation; or preparation of the manuscript.

## Ethics Statement

This study was conducted in accordance with applicable ethical principles and was approved by the Ethics Committee of the Second Affiliated Hospital of Harbin Medical University (Approval No. KY2026‐057). All participants were informed of the study’s purpose and procedures and provided written informed consent prior to participation. Confidentiality was maintained, and participation was entirely voluntary.

## Consent

Please see the Ethics Statement.

## Conflicts of Interest

The authors declare no conflicts of interest.

## Supporting Information

Additional supporting information can be found online in the Supporting Information section.

## Supporting information


**Supporting Information** Supporting Table S1. Parametric coefficients from the generalized additive model predicting professional commitment, including adjusted linear effects of demographic covariates with estimates, standard errors, test statistics, *p* values, and 95% confidence intervals. Supporting Table S2. Predictor importance based on permutation analysis using the iml framework, showing the relative contribution of demographic and psychosocial predictors to model prediction performance. Supporting Figure S1. Exploratory conditional patterns between workplace anxiety and professional commitment across representative levels of spiritual leadership, with model‐estimated predictions and 95% confidence intervals. STROBE checklist.

## Data Availability

The datasets used and/or analyzed in this study are available from the corresponding author upon reasonable request (Yuhuan Zhang, 2802262584@qq.com).
