# Supplementary material for: Psychological and Organizational Factors Associated With Professional Commitment Among Newly Graduated Psychiatric Nurses: Exploring the Roles of Workplace Anxiety, Spiritual Leadership, and Error Management Climate
Source: J Nurs Manag. 2026 Aug 31;2026:5696980. doi: 10.1155/jonm/5696980 (PMC13530871; doi:10.1155/jonm/5696980)
Supplement: Supplementary file 1 [file JONM-2026-5696980-s001.zip › Brief Additional Description of Supplementary Mate.docx]

**Brief Additional Description of Supplementary Material**

Supplementary Table S1. Parametric coefficients from the generalized additive model predicting professional commitment, including adjusted linear effects of demographic covariates with estimates, standard errors, test statistics, p values, and 95% confidence intervals.

Supplementary Table S2. Predictor importance based on permutation analysis using the iml framework, showing the relative contribution of demographic and psychosocial predictors to model prediction performance.

Supplementary Figure S1. Exploratory conditional patterns between workplace anxiety and professional commitment across representative levels of spiritual leadership, with model-estimated predictions and 95% confidence intervals.
