# Supplementary material for: Psychological and Organizational Factors Associated With Professional Commitment Among Newly Graduated Psychiatric Nurses: Exploring the Roles of Workplace Anxiety, Spiritual Leadership, and Error Management Climate
Source: J Nurs Manag. 2026 Aug 31;2026:5696980. doi: 10.1155/jonm/5696980 (PMC13530871; doi:10.1155/jonm/5696980)
Supplement: Supplementary file 1 [file JONM-2026-5696980-s001.zip › Supplementary Figure S1.docx]

**Supplementary Figure S1.Exploratory conditional patterns between workplace anxiety and professional commitment across levels of spiritual leadership**

Note:Predicted professional commitment scores across the observed range of workplace anxiety are presented at three representative levels of spiritual leadership (30, 50, and 70 points). Solid lines indicate model-estimated conditional predictions, and shaded areas represent 95% confidence intervals. The figure illustrates potential variation in the relationship between workplace anxiety and professional commitment according to different levels of spiritual leadership. Although the negative association between workplace anxiety and professional commitment appeared weaker at higher levels of spiritual leadership, the workplace anxiety × spiritual leadership interaction did not remain statistically significant after Bonferroni correction (adjusted p = 0.099). Therefore, these patterns should be interpreted as exploratory and not considered confirmatory evidence of moderation.
