# Supplementary material for: Psychological and Organizational Factors Associated With Professional Commitment Among Newly Graduated Psychiatric Nurses: Exploring the Roles of Workplace Anxiety, Spiritual Leadership, and Error Management Climate
Source: J Nurs Manag. 2026 Aug 31;2026:5696980. doi: 10.1155/jonm/5696980 (PMC13530871; doi:10.1155/jonm/5696980)
Supplement: Supplementary file 1 [file JONM-2026-5696980-s001.zip › Supplementary Table S1.docx]

**Supplementary Table S1. Parametric coefficients from the generalized additive model predicting professional commitment**

| **Predictor** | **Estimate** | **Standard Error** | **t value** | **p value** | **95% CI** |
| --- | --- | --- | --- | --- | --- |
| Intercept | 91.67 | 9.82 | 9.34 | <0.001 | 72.43 to110.91 |
| Age | -0.03 | 0.38 | -0.07 | 0.946 | -0.77 to 0.71 |
| Sex (Female vs Male) | 1.36 | 1.65 | 0.83 | 0.408 | -1.86 to 4.59 |
| Only-child status (No vs Yes) | -0.46 | 1.59 | -0.29 | 0.774 | -3.57 to 2.66 |
| Marital status (Married vs Unmarried) | 0.89 | 1.63 | 0.55 | 0.584 | -2.30 to 4.09 |
| Education (Master or above vs Bachelor or below) | 0.7 | 2.21 | 0.32 | 0.751 | -3.63 to 5.03 |

**Note:** Parametric coefficients represent adjusted linear effects of demographic covariates included in the generalized additive model. Estimates are presented with approximate 95% confidence intervals calculated from the covariance matrix of the fitted model. Confidence intervals crossing zero indicate that the corresponding covariate effects were not statistically significant at the 0.05 level.
