# Supplementary material for: Psychological and Organizational Factors Associated With Professional Commitment Among Newly Graduated Psychiatric Nurses: Exploring the Roles of Workplace Anxiety, Spiritual Leadership, and Error Management Climate
Source: J Nurs Manag. 2026 Aug 31;2026:5696980. doi: 10.1155/jonm/5696980 (PMC13530871; doi:10.1155/jonm/5696980)
Supplement: Supplementary file 1 [file JONM-2026-5696980-s001.zip › Supplementary Table S2.docx]

**Supplementary Table S2. Predictor Importance Based on Permutation Analysis Using the iml Framework**

| **Predictor** | **Mean \|Importance\|** |
| --- | --- |
| Error management climate | 5.031 |
| Workplace anxiety | 3.817 |
| Spiritual Leadership | 2.442 |
| Age | 1.654 |
| Only Child | 1.183 |
| Marital Status | 1.176 |
| Sex | 1.153 |
| Education | 1.122 |

Permutation-based variable importance analysis was used to quantify the relative contribution of each predictor to the model's predictive performance. Error management climate showed the highest permutation importance, followed by workplace anxiety and spiritual leadership. Demographic variables demonstrated comparatively smaller contributions
