# Supplementary material for: Psychological and Organizational Factors Associated With Professional Commitment Among Newly Graduated Psychiatric Nurses: Exploring the Roles of Workplace Anxiety, Spiritual Leadership, and Error Management Climate
Source: J Nurs Manag. 2026 Aug 31;2026:5696980. doi: 10.1155/jonm/5696980 (PMC13530871; doi:10.1155/jonm/5696980)
Supplement: Supplementary file 1 [file JONM-2026-5696980-s001.zip › STROBE.pdf]

Reporting checklist for cross sectional study.

Based on the STROBE cross sectional guidelines.

Instructions to authors

Complete this checklist by entering the page numbers from your manuscript where readers will find each of the items listed below.

Your article may not currently address all the items on the checklist. Please modify your text to include the missing information. If you are certain that an item does not apply, please write "n/a" and provide a short explanation.

Upload your completed checklist as an extra file when you submit to a journal.

In your methods section, say that you used the STROBE cross sectionalreporting guidelines, and cite them as:

von Elm E, Altman DG, Egger M, Pocock SJ, Gotzsche PC, Vandenbroucke JP. The Strengthening the Reporting of Observational Studies in Epidemiology (STROBE) Statement: guidelines for reporting observational studies.

		Reporting Item	Page Number	Notes / Explanation
**Title and abstract** Title	#1a	Indicate the study's design with a commonly used term in the title or the	2	The abstract states "cross-sectional study" in ; the title itself describes the study design
Abstract	#1b	abstract Provide in the abstract an informative and balanced summary of what was	2	Abstract includes background, objective, methods, results, and conclusions with key numerical findings
**Introduction** Background /rationale	#2	done and what was found Explain the scientific background and rationale for the investigation being reported	5-7	Background covers nursing shortages, psychiatric nursing challenges, transition shock, and the research gap regarding workplace anxiety, spiritual leadership, and error management climate
Objectives	#3	State specific objectives, including any prespecified hypotheses	8	Objectives clearly stated: "to investigate the associations and potential interactions among workplace anxiety, spiritual leadership, error management climate, and professional commitment"

**Methods**<br>Study design	#4	Present key elements of study design early in the paper	8	Stated as "cross-sectional methodology" at the start of Methods section
Setting	#5	Describe the setting, locations, and relevant dates, including periods of	8,12	Three tertiary psychiatric institutions in Tianjin, China described; data collection timeframe mentioned in 2.4 (online survey through Wenjuanxing)
Eligibility criteria	#6a	recruitment, exposure, follow-up, and data collection<br>Give the eligibility criteria, and the sources and methods of selection of	9	Inclusion and exclusion criteria clearly listed; convenience sampling method stated
	#7	participants.<br>Clearly define all outcomes, exposures, predictors, potential confounders, and	10-12	All variables (workplace anxiety, spiritual leadership, error management climate, professional commitment) defined in 2.3; demographic covariates listed
Data sources /measurement	#8	effect modifiers. Give diagnostic criteria, if applicable<br>For each variable of interest give sources of data and details of methods of assessment (measurement). Describe comparability of assessment methods if there is more than one group. Give information separately for for exposed and unexposed groups if applicable.	10-12	Each scale described with developer information, number of items, scoring, and reliability coefficients (Cronbach's α, KMO, Bartlett's test)

Bias	#9	Describe any efforts to address potential sources of bias	13	Exclusion of invalid responses (completion time <2 min, identical responses) described in 2.4; limitations discuss recall bias, social desirability bias, and common method bias in Section 6
Study size	#10	Explain how the study size was arrived at	9	Green's rule ($N \geq 50 + 8m$) explained; final sample of 264 exceeds minimum of 114; rationale for GAM and RF sample adequacy discussed
Quantitative variables	#11	Explain how quantitative variables were handled in the analyses. If applicable,describe which groupings were chosen, and why	13-14	Continuous variables summarized using means and SDs; categorical variables converted to factors; age, sex, only-child status, marital status, educational attainment included as linear covariates
Statistical	#12a	Describe all statistical methods, including those used to control for confounding	14	GAM detailed (thin-plate splines, REML estimation, basis dimension k=4); RF described (ranger algorithm, 10-fold CV, 500 trees); demographic covariates adjusted in GAM
methods Statistical	#12b	Describe any methods used to examine subgroups and interactions	14	Tensor-product smooths "te()" applied for prespecified pairwise interactions; Bonferroni correction for multiple interaction testing
methods Statistical	#12c	Explain how missing data were addressed	13	"Data completeness was verified prior to analysis, and no missing data imputation was required"
methods Statistical	#12d	If applicable, describe analytical methods taking account of sampling strategy	NA	Convenience sampling was used; no sampling weights applied; not applicable
methods Statistical	#12e	Describe any sensitivity analyses	16	Bootstrap resampling (500 iterations) for stability analysis; repeated 10-fold cross-validation for RF tuning

methods<br>**Results**<br>Participants	#13a	Report numbers of individuals at each stage of study—eg numbers potentially	13<br>6	290 questionnaires received, 26 invalid excluded, 264 retained; effective response rate 91.03%
Participants	#13b	eligible, examined for eligibility, confirmed eligible, included in the study, completing follow-up, and analysed. Give information separately for for exposed and unexposed groups if applicable.<br>Give reasons for non-participation at each stage	13	Reasons for exclusion: completion time <2 minutes, identical responses across all items; clearly stated in 2.4
Participants	#13c	Consider use of a flow diagram	NA	recruitment and screening process was straightforward (290 received → 26 excluded → 264 retained), and clearly described in text (subsection 2.4). Textual description is sufficiently transparent; a flow diagram would add redundancy without additional value.
Descriptive data	#14a	Give characteristics of study participants (eg demographic, clinical, social) and	17	Table 1 presents all demographic characteristics and key study variables (means and SDs)
Descriptive data	#14b	information on exposures and potential confounders. Give information separately for exposed and unexposed groups if applicable.<br>Indicate number of participants with missing data for each variable of interest	13	"Data completeness was verified prior to analysis, and no missing data imputation was required"
Outcome data	#15	Report numbers of outcome events or summary measures. Give information	17	Professional commitment summary measure (mean = 92.27, SD = 16.39) reported in Table 1
Main results	#16a	separately for exposed and unexposed groups if applicable.<br>Give unadjusted estimates and, if applicable, confounder-adjusted estimates	19	GAM smooth terms (F, p-values, EDF) presented in Table 2; adjusted for age, sex, only-child status, marital status, educational attainment; CI for parametric coefficients noted in 3.2.2 ("all 95% CIs included zero")

Main results	#16b	and their precision (eg, 95% confidence interval). Make clear which confounders were adjusted for and why they were included<br><br>Report category boundaries when continuous variables were categorized	NA	Continuous variables were not categorized in the primary analyses; treated as continuous in GAM and RF
Main results	#16c	If relevant, consider translating estimates of relative risk into absolute risk for a	NA	Not applicable for cross-sectional study with continuous outcome
Other analyses	#17	meaningful time period<br><br>Report other analyses done—e.g., analyses of subgroups and interactions, and	19,20-21,25	Interaction analyses (three tensor-product smooths) reported in Table 2; exploratory interaction analysis described in 3.2.3; bootstrap stability analysis in 3.5
**Discussion**<br><br>Key results	#18	sensitivity analyses<br><br>Summarise key results with reference to study objectives	26	Opening paragraph summarises the four principal findings in relation to the study objectives

Limitations	#19	Discuss limitations of the study, taking into account sources of potential bias or imprecision. Discuss both direction and magnitude of any potential bias.	33	Four limitations discussed: single-city sampling, self-reported measures (recall bias, social desirability, common method bias), cross-sectional design (causality), and sample size/model complexity
Interpretation	#20	Give a cautious overall interpretation considering objectives, limitations,	26-32	Section 4 provides cautious interpretatio[illegible] strength theory, social exchange the[illegible] interpret[illegible]
Generalisability	#21	multiplicity of analyses, results from similar studies, and other relevant evidence. Discuss the generalisability (external validity) of the study results	33	Addressed in Limitations: participants from three Tianjin institutions may limit generalisability; future multi-center studies needed
**Other Information** Funding	#22	Give the source of funding and the role of the funders for the present study and, if applicable, for the original study on which the present article is based	36	Funding sources are listed (24GJZXE003 and HYDSZZDXM011). The funders had no role in study design, data collection, analysis, interpretation, or manuscript preparation. This is an original study; no prior study is being reported.

The STROBE checklist is distributed under the terms of the Creative Commons Attribution License CC-BY. This checklist was completed on 31. March 2023 usinghttps://www.goodreports.org/, a tool made by the[EQUATOR Network](https://www.equator-network.org/)in collaboration with[Penelope.ai](https://www.penelope.ai/)
